# Identification of Essential Features in Developing a Novel Femoral Stem Reflecting Anatomical Features of East Asian Population: A Morphological Study

**DOI:** 10.3390/jcm13206030

**Published:** 2024-10-10

**Authors:** Ji Hoon Bahk, Seung-Beom Han, Kee Hyung Rhyu, Jeong Joon Yoo, Seung-Jae Lim, Kwan Kyu Park, Sang-Min Kim, Young Wook Lim

**Affiliations:** 1 Department of Orthopedic Surgery, Bucheon St. Mary’s Hospital, College of Medicine, The Catholic University of Korea, Seoul 14647, Republic of Korea; tonybahk@gmail.com; 2 Department of Orthopedic Surgery, Korea University Medical Center, College of Medicine, Korea University, Seoul 30019, Republic of Korea; oshan@korea.ac.kr; 3 Department of Orthopedic Surgery, Kyung Hee University Hospital, College of Medicine, Kyung-Hee University, Seoul 02447, Republic of Korea; khrhyu@gmail.com; 4 Department of Orthopedic Surgery, Seoul National University Hospital, College of Medicine, Seoul National University, Seoul 03085, Republic of Korea; jjyos@snu.ac.kr; 5 Department of Orthopedic Surgery, Samsung Medical Center, College of Medicine, Sungkyunkwan University, Seoul 06351, Republic of Korea; limsj70@gmail.com; 6 Department of Orthopaedic Surgery, Severance Hospital, College of Medicine, Yonsei University, Seoul 03722, Republic of Korea; kkpark@yuhs.ac; 7 Department of Orthopaedic Surgery, Korea University Guro Hospital, College of Medicine, Korea University, Seoul 30019, Republic of Korea; 8 Department of Orthopedic Surgery, Seoul St. Mary’s Hospital, College of Medicine, The Catholic University of Korea, Seoul 06591, Republic of Korea

**Keywords:** femoral stem, total hip arthroplasty, metaphyseal–diaphyseal mismatch, stem design, fit analysis

## Abstract

**Background**: Recent advancements in hip arthroplasty aim to enhance the stability, longevity, and functionality of femoral implants. However, the distal fitting of femoral stems, often caused by metaphyseal–diaphyseal mismatch, remains a significant issue, particularly in patients with Dorr type A femora. Such mismatches can result in suboptimal implant performance, leading to potential complications. This study focuses on evaluating the anatomical compatibility of five representative single-tapered wedge mid–short stems with the mediolateral (ML) anatomy of the proximal femur in an East Asian population, where these mismatches are often more pronounced. **Methods**: A total of 742 patients from two hospitals, all of whom underwent unilateral primary total hip arthroplasty, were included in the study. The contralateral proximal femur was confirmed to have normal anatomy in each patient. Hip anteroposterior radiographs were used for measurements, which were standardized in conjunction with CT images. Key anatomical parameters were measured, including proximal and distal medial–lateral canal dimensions, vertical offset, and medial offset. Five femoral stem designs—Tri-lock^®^, Taperloc^®^, Anthology^®^, Accolade II^®^, and Fit^®^—were evaluated. R programming was employed for a detailed fit analysis to match stem sizes with patient anatomy, categorizing the fit as proximal, simultaneous proximal–distal, or distal engagement. **Results**: Among the femoral stems analyzed, the Fit^®^ stem demonstrated the closest alignment with the regression line for ML widths in the study population (slope = 0.69; population ML slope = 0.38). This was followed by Accolade II^®^, which had a slope of 0.83. In terms of offset options, the Accolade II^®^ offered the largest offset coverage, making it particularly suitable for this population. The fit analysis revealed that the Fit^®^ stem had the highest suitable fit rate (90.56%), followed by Accolade II^®^ (73.04%). Taperloc^®^, Anthology^®^, and Tri-lock^®^ had similar fit rates of approximately 59%. Overall, optimal results were obtained for 92.05% of the population in the automated fitting trial, regardless of the product type. **Conclusions**: When designing modern cementless femoral stems intended for press-fit fixation, it is crucial to account for the anatomical variations specific to the target population. In this study, Fit^®^ and Accolade II^®^ femoral components demonstrated superior compatibility with the femoral anatomy of the East Asian population, particularly in those with a higher incidence of Dorr type A femora. These stems, characterized by slimmer distal dimensions and high-offset options, appear to minimize metaphyseal–diaphyseal mismatch and associated complications.

## 1. Introduction

Hip arthroplasty is one of the most successful advances in modern surgical treatment. Femoral implants have been evolving to increase stability, longevity, and function so as to overcome complications including persistent thigh pain, which is reported to be 3.8–16.7% [1,2,3,4] for cementless stems. Thigh pain is still an area of concern, which is known to be more frequent in patients with Dorr type A [5,6] proximal femora, possibly due to higher chances of distal engagement of the stem or metaphyseal–diaphyseal mismatch [5,7,8,9]. The phenomenon is directly associated with stem design and configuration.

Proximal femur geometry is the fundamental basis for developing the femoral component. It is crucial in implant stability and osseointegration potential, which directly affects longevity, especially for cementless stems, as they require press-fitting by precise figures in near-exact correspondence to the anatomy of the proximal femur. Inappropriate vertical femoral offset after surgery results in leg length discrepancy, whereas decreased medial offset would be liable for dislocation due to decreased soft-tissue tension. Increased medial offset brings about higher tension in the hip abductors, which is a known factor of trochanteric bursitis.

Dorr et al. classified proximal femora into the following three types based on the cortical thickness measured 10 cm below the mid-level of the lesser trochanter [6]: Type A—characterized by a thick cortex with a narrow diaphyseal canal; Type B—exhibiting moderate cortical thickness and canal width, with medial and posterior cortical bone loss; and Type C—showing dramatic cortical thinning, resulting in a very wide intramedullary canal. Distal fitting of the stem can cause distressful results of leg lengthening or relative downsizing of the femoral component [10,11,12], which is a more frequent phenomenon in the Asian population, who possess a relatively high femoral cortical index or Dorr type A femora [5,13]. The mismatch is suspected to result in a higher rate of overall complications in Dorr type A than in type B femora [5].

However, more frequently used commercially available shortened standard stems from major manufacturers were developed based on the average anatomy of the Western population. These designs might not be as well of a fit for the Asian population [6,14,15]. Despite the efforts to reflect the anatomical characteristics of the sample population, variation among ethnicity can act directly as a major factor of differences in complication rates. In this study, analyses for the compatibility of five representative single-tapered wedge mid–short stems to the sample population were performed by matching mediolateral (ML) widths and offsets by a computed simulation trial through coding. By searching for the best fitting stem for the study population, the aim was to elucidate the key features an improved stem might possess.

## 2. Materials and Methods

### 2.1. Patient Selection

Patients who underwent unilateral primary total hip arthroplasty between March 2015 and March 2020 with a disease-free and undeformed contralateral hip joint, as well as normal anatomy of the proximal femur, characterized by a physiologic neck–shaft angle (120°–135°) [16], were included in the study. Hips with a documented history or radiographic traces of prior fracture, sequelae resulting from any disease, and pediatric hips below the age of 18 were excluded from the sample selection. A total of 344 patients from one university hospital and 400 patients from another university hospital of a different institute were assessed. In total, plain anteroposterior (AP) hip radiographs from 742 patients were included to be reviewed by four orthopedic surgeons. The sample size was determined to be sufficient for linear regression (*n* ≥ 48) based on an a priori power analysis with a power of 95% (G*Power version 3.1.9.4., Kiel, Germany). The demographics of the study population are delineated in Table 1. This study was approved, and the consent was waived by the Institutional Review Board of the two university hospitals.

### 2.2. Radiographic Standardization

We utilized routinely taken hip anteroposterior preoperative templating radiographs and 3D pelvic bone computed tomography (CT) under quality control for radiographic measurements for all patients. To obtain a true AP view of the proximal femur, templating films were taken in the AP view of the pelvis centered over the pubic symphysis during manual internal rotation (10° to 15°) applied to the contralateral normal hip by the examiner [17]. To minimize discrepancies occurring by magnification errors, a coin that measures 26.5 mm in diameter was taped to the level of the hip joint for each radiograph, which was originally described by Oddy et al. [18] using a ten-pence coin. The coin was used as a reference of scale for the estimation of real distance using the picture archiving communication system (PACS) measuring tool (nU PACS version 1.0.0.42.1, Anyang, Republic of Korea).

To validate the accuracy and reproducibility of measurements using digital radiographs via the PACS, two investigators measured the medial offsets (distance between the femoral head center and the anatomical femoral axis) from 20 randomly selected samples which were repeated in two-week intervals. The measurements were conducted for both templating AP digital film and its corresponding isolated proximal femur 3D reconstruction CT series in the same view for each patient. The average absolute difference was 0.76 mm (SD, 0.57), ranging from 0.07 to 2.26 mm. No significant difference was found in measurement methods using templating AP film (Ysio MAX 3D, Siemens Healthcare, Erlangen, Germany) and CT (SOMATOM Force, Siemens Healthineers, Erlangen, Germany) with a power of 0.999 (n = 20, α = 0.05) by a paired *t*-test (*p* = 0.688), and the intraclass correlation coefficient (ICC) for absolute agreement between the two methods was 0.993 (*p* = 0.000). The ICCs for consistency in inter-observer and intra-observer reliability were 0.869 and 0.948, respectively (*p* = 0.000).

### 2.3. Radiographic Measurements

**Figure 1:**
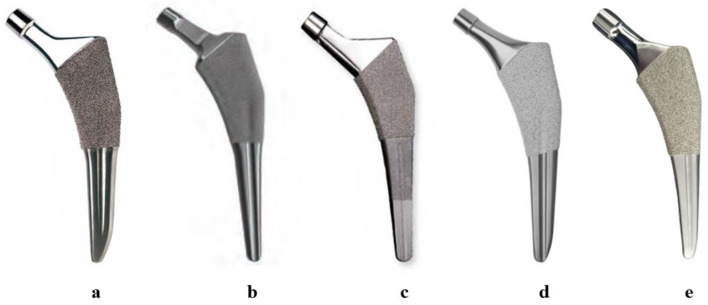
Commercialized femoral stems from five different manufacturers, which were included in the analysis. (**a**) Depuy Tri-lock^®^, (**b**) Biomet Taperloc^®^, (**c**) Smith & Nephew Anthology^®^, (**d**) Stryker Accolade II^®^, (**e**) Corentec Fit^®^.

Measurements were carried out from the normal hip joint for all patients. Obtained key parameters included the proximal–medial canal size, distal canal size, vertical offset, and medial offset of the proximal femur. The proximal–medial ML width was determined as the distance of the medial gap between the anatomical proximal femoral axis and the inner medial cortex at a 10 mm vertically proximal level from the tip of the lesser trochanter (LT). The distal ML width was set as the canal diameter between the inner cortices at a 60 mm distal level from the tip of the LT [14,19,20] (Figure 1).

For femoral stems as the counterpart, the medial starting point of the proximal porous coating for each stem was used as the reference point of measurement. Assuming that stem alignment is neutral, the proximal–medial ML width and distal ML widths were measured at 20 mm and 80 mm distal levels from the reference point, respectively. Vertical offsets (or neck height of an implant) and medial offsets were measured from the center of the femoral head to the level of the reference point and anatomical femoral axis, respectively. The anatomical axis of the femur shaft was drawn by connecting the midpoints of one horizontal line at the caudal tip of the LT and a second line more caudally at the femoral diaphysis. All measurements for implant specifications were conducted referring to product brochures and on templating films, not on the radiograph.

### 2.4. Femoral Stems for Evaluation

**Figure 2:**
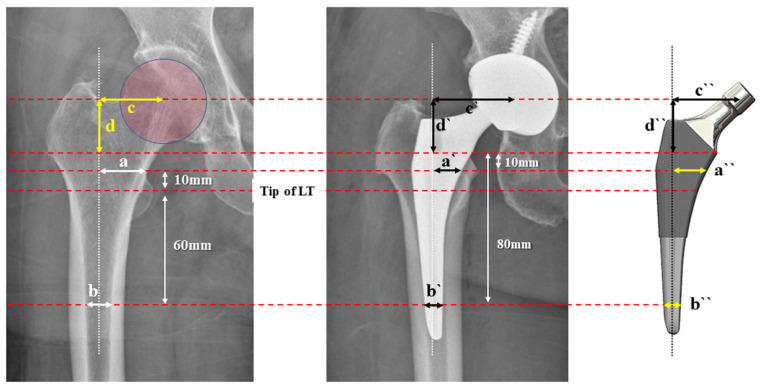
Reference points of measurement in this study. For radiographic evaluation, proximal–medial ML width was measured at 10 mm proximal to the tip of the LT (a) and distal ML width was measured at 60 mm distal (b). For implant parameters, a 10 mm distal level to the medial proximal starting point of the porous coating was used to measure proximal–medial ML width (a`) and an 80 mm distal level for distal ML width (b`). The medial offset (c,c`) and vertical offset (d,d`) were also measured for the offset analysis. Corresponding parameters are also shown on the schematic illustration of a femoral implant on the right (a``–d``).

The recent top American market shares of femoral components for hip arthroplasty are mostly occupied by single-tapered wedge (type I), proximal-coated, mid–short-sized cementless stems [21]. Among the type I stems, we selected five representative stems from five different manufacturers for analyses in the current study, namely Tri-lock (DePuy Orthopaedics, Inc., Warsaw, IN, USA), Taperloc (Biomet Orthopedics, Warsaw, IN, USA), Anthology (Smith and Nephew Inc., Memphis, TN, USA), Accolade II (Stryker Orthopaedics, Mahwah, NJ, USA), and Core-fit (Corentec, Seoul, Republic of Korea). Fit is a recently developed type I femoral stem designed for a more tailored fit for the Asian population. Measurements from all available conventional stem sizes and offset options supplied by the manufacturers were analyzed in the study (Figure 2, Table 2).

### 2.5. ML Width Analysis

Results of the regression analysis were plotted in discrete lines for each of the five stems, which were compared with the linear regression line derived from the whole background data obtained from the study sample population. Background data were depicted as separate dots, each representing a patient. Then, their regression line was delineated as a thick line with an adjacent gray zone indicating the 5% standard deviation. The slope of the proximal–medial ML width to the distal ML width was compared between the study population and the femoral stems.

### 2.6. Offset Analysis

The measurements of vertical and medial offsets were represented as background dots and their regression line was delineated with an adjacent gray zone indicating the 5% standard deviation. Standard offset (132° for Accolade II) and high offset (127° for Accolade II) options were separately presented. Furthermore, typical neck length options including short, medium, and long were also indicated, with each option entailing a distinct set of offset lengths.

Subsequently, the relative coverage area of the study population for each stem was calculated for the comparison of stem offset compatibility. The calculation was employed using the R package ggplot2 (version 3.3.5) by determining the value of the accumulative surface area (mm^2^) of the overall vertical and medial offsets of a specific series of a femoral stem, which can theoretically be provided through utilizing their variable neck length and offset options.

### 2.7. Fit Analysis Using R Coding

**Figure 3:**
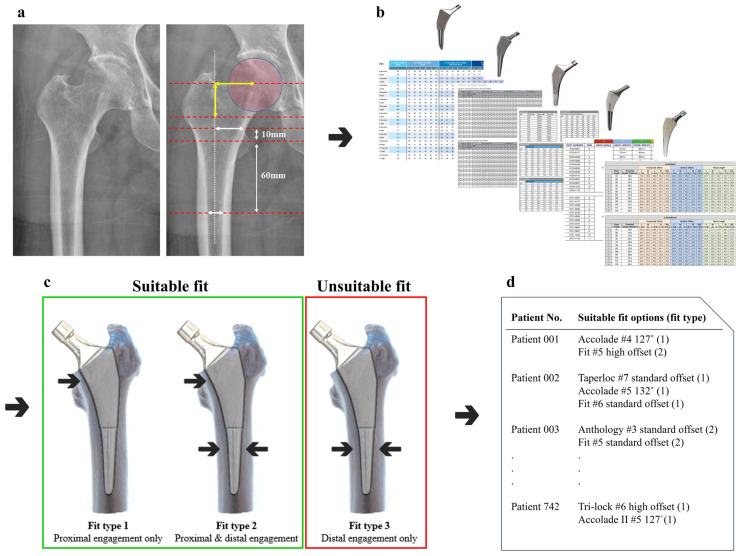
A flow diagram outlining the fit analysis process was implemented. (**a**) Anatomical parameters extracted from the radiographs of each patient were encoded with R for matching trials encompassing (**b**) all available sizes from the five femoral stem products. (**c**) Fit types 1 and 2 were deemed suitable, while type 3 or unmatched samples were categorized as unsuitable. (**d**) Through the consolidation of outputs that integrated specific suitable fit options for each patient, the results were structured for the comparative assessment of the subject femoral stems.

Automated trials were coded to match all available sizes of femoral stems for each patient, as illustrated in Figure 3. In each trial, the extracted anatomical parameters of each patient were collated with femoral stem parameters one by one to provide a binary output, delineating either a suitable or unsuitable fit. The output of each femur was classified as fit type 1 (proximal engagement only), 2 (simultaneous proximal and distal engagement), or 3 (distal engagement only). Fit types 1 and 2 were considered as fit or suitable for each patient, and type 3 as unsuitable. A value of 0.5 mm for under-fitting and 1.0 mm for over-fitting was considered acceptable considering possible variations in actual implantation sizes and its possible influences by rasp control. An under-fitting of a femoral component denotes a situation where the fit is smaller than the exact anatomical ML width, whereas over-fitting refers to a fit that is larger than the exact anatomical ML width. We coded a classify_fit function to automate the large-scale data process of comparing each measurement from different stem sizes by calculating the differences and checking if they fell within predefined thresholds. The results were then aggregated and visualized using the geom_bar function.

To ascertain comprehensive outcomes by determining the most optimal type of femoral component, along with its appropriate size for each patient, preference was given to the selection of fit type 1 stems over type 2 stems when both types were deemed suitable. This prioritization was based on the ideal design of the subject stems as proximal fitting stems. The results were integrated for a comparative fit assessment of the femoral stems, and their statistical difference was additionally verified by Pearson chi-square analysis. The classification process was implemented using R (version 3.6.3, R Project for Statistical Computing, Vienna, Austria). All statistical analyses, coding, and plotting were conducted using R with ggplot2 packages.

## 3. Results

### 3.1. ML Width Analysis

**Figure 4:**
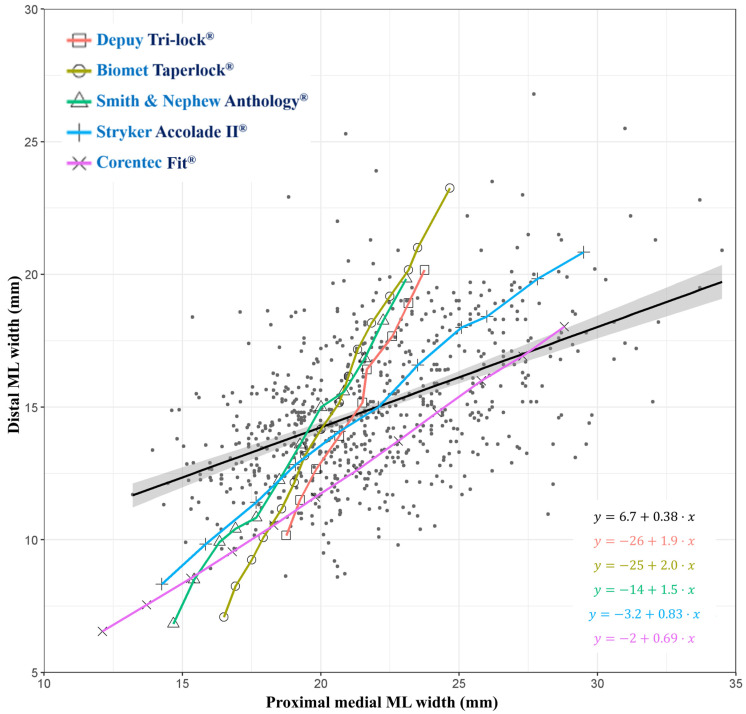
Plots as a result of ML width analysis. The regression line from background dots of individuals in the study population is depicted as a thick black line with a 95% confidence interval as shaded areas. Distal ML widths to proximal–medial ML widths are shown for five femoral stems, with slopes calculated and presented in the lower right corner.

A stem that exhibits the closest slope to the regression line of the study population was considered to provide the best coverage for the population’s anatomical variability. The slope of Fit (0.69), followed by Accolade II (0.83), showed the best proximity to the slope of the study population (0.38). Anthology (1.50), Tri-lock (1.90), and Taperloc (2.00) exhibited higher slopes, missing coverage of patients with a longer proximal–medial ML width in association with a shorter distal ML width (Figure 4).

### 3.2. Offset Analysis

**Figure 5:**
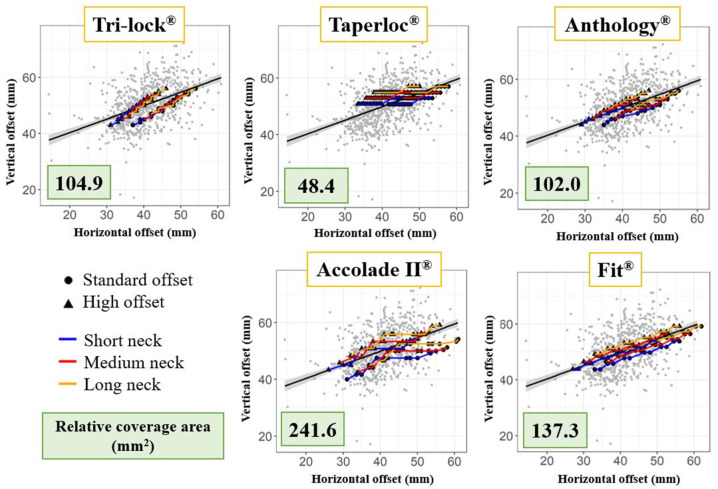
Separate plots for offset analyses for each stem. Offset options and neck options are accumulatively depicted in the plot. The relative coverage area is calculated by R for comparison among stems.

Plots using vertical-to-medial offset for each stem were separately depicted on the background data of the study population (Figure 5). Tri-lock, Anthology, and Fit showed a relatively consistent slope by increasing stem sizes while also offering high medial offset options with patterns of parallel translation. It is noteworthy that Accolade II shows a flat section in the mid-size stems where the medial offset increases without increasing the vertical offset, providing wider coverage for the population. Taperloc possesses a characteristically different pattern of exhibiting two long sections of fixed vertical offsets with only the medial offsets increasing.

In terms of relative coverage, which indirectly shows how broadly a stem can cover within the study population, Accolade II offered the largest area, followed by Fit, Tri-lock, Anthology, and Taperloc. Fit exhibited the closest trend to the regression line of the population, but the high offset option only provided a relatively small coverage area.

### 3.3. Fit Analysis

**Figure 6:**
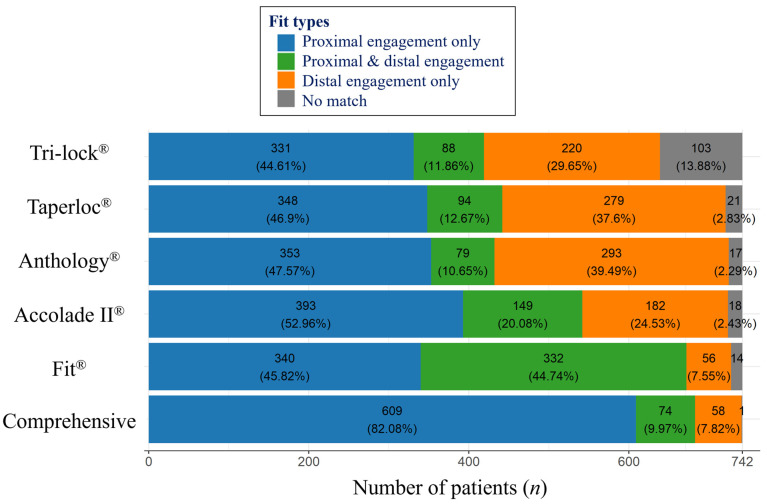
Fit type analysis using the automated trials of all available sizes of each stem. Integrated results of determining the most optimal result for an individual are shown as ‘overall’. Fit analysis was performed by R, coding the automated trial of all available options and sizes of the stems. A 0.5 mm under-fitting and 1.0 mm over-fitting was permitted to print out the suitability for the dataset of anatomical measurements of each patient.

For the fitting analysis by the trial of all available sizes of each stem, utilizing data from the ML width analysis, Fit (90.56%) best provided suitable stems for the patients with significance (*p* < 0.001, Pearson chi-square), followed by Accolade II (73.04%). Among the suitable results, the proportion of fit type 2 (simultaneous proximal and distal engagement) made a difference between stems. Taperloc (59.57%), Anthology (58.22%), and Tri-lock (56.47%) showed a similar fit for this population. The overall results of the automated fitting trial, regardless of the product type, showed that optimal results were obtained for 92.05% of the population (Figure 6).

## 4. Discussion

Distal engagement of cementless femoral stem lacking proper proximal fixation is a remaining challenge for improvement in total hip arthroplasty. Considerations of anatomical characteristics have become essential in developing newer femoral stems, in that modern cementless stems solely rely on press-fit fixation. Existing ready-made stems generally fit most of the population but for some ethnic groups, but unsuitable fixation tends to occur in more cases. Therefore, the importance of big data-based stem development that reflects the anatomical features of the population is more emphasized. For example, the Accolade II femoral stem was developed utilizing the SOMA (Stryker Orthopaedics Modelling and Analytics Technology) database, reflecting a large amount of anatomical data of demographic characteristics [22], which led us to investigate the essential features that a newer stem must possess, yet the database only contained 16% of Asian femora [23].

The reference points for measurement were mostly determined by referring to other studies [19,22], but some modifications were made for implant measurements. Proximal–medial ML widths were determined at a 10 mm proximal level from the tip or the midpoint of the LT as in other references. But for the measurement of proximal–medial ML widths in femoral stems, a 10 mm distal level from a different reference line—the medial starting point of the porous coating—was used due to the following reasons: (1) the neck cutting level is commonly targeted 5 to 10 mm above the most proximal point of the LT and (2) the starting point of the porous coating on the medial side of the stem is the target guide for the ideal insertion depth. Hence, we believed that the length of the LT itself plus the length of the remnant neck should be considered when setting the anatomical point for comparison between implants.

Upon analyzing ML widths, stems with a lower distal ML width-to-proximal–medial ML width slopes offered a wider coverage of the population, but stems with higher slopes did not tend to fit patients who possess a longer proximal–medial ML width in association with a shorter distal ML width or Dorr type A femora. The slopes translate into how well the gradually increasing distal thickness of each stem aligns with the Asian population with relatively thicker mean distal cortices (higher FCIs). That is, stems with lower slopes may provide a better fit when considering the characteristics of the sample population. Accordingly, Anthology, Tri-lock, and Taperloc might have a higher chance of distal-only engagement, intraoperative periprosthetic fracture, or thigh pain in patients with a higher FCI.

A comparison of the relative coverage area among various offset and neck length options does not directly signify how many femora are compatible to a stem. Instead, it represents a notion of a specific stem’s capability to cope with various anatomical situations. The results suggest that offering high offset options are essential for widening the coverage within the population, but also the gap between offset options must not be too small. High offset options of Accolade II provided not only lateral-to-lateral shift but also variations in vertical offsets by adjusting the caput–collum–diaphyseal (CCD) angles, thus achieving the largest coverage.

The measurement data and the results for fit analysis imply that stem designs with slimmer distal ML widths have a lesser frequency of metaphyseal–diaphyseal mismatch, minimizing the risk of complications. Moreover, patients with suboptimal results should not be concluded, as none of the stem options are available. We set an adequate standard of permitting over-fitting, which can be sufficiently surmounted by the surgeon’s rasp control and implant insertion skills. In fact, distal-only engagement fit types in this simulation generally would undergo hip arthroplasty without serious obstacles.

There are several limitations to this study. Anteroposterior three-point fixation is an important factor for a stem design, which can be reviewed by a CT scan or well-taken trans-lateral hip radiographs. Furthermore, femoral components with a shorter AP width may establish engagement in the AP direction before achieving an ML fit. But we limited this study to only mediolateral evaluation with single-tapered wedge, mid–short-sized cementless products to emphasize the structural tendency for distal engagement, which generally occurs in the coronal plane. Thus, all analyses in this study assumed only the optimal final press-fit position of a femoral component, without taking into consideration the trajectory of inserting the implant, which can be critical in the surgical procedure [24]. In defining the fit that judges the adequacy of fixation, under-fitting and over-fitting were permitted arbitrarily based on generally well-fixed stems, and simple adjustments are always possible through rasp control. To our knowledge, there has been no literature that investigated the standards of an optimal fit for the metaphyseal and diaphyseal contact gap. When considering the valgus or varus alignment of an inserted stem in acceptable degrees, it would be relatively more acceptable in real circumstances. Accordingly, subsequent analyses are readily possible using different gap allowances using the matching platform set by coding.

There is a potential bias in the selection of specific femoral components included in this study, and further research with newer or alternative stems may yield different results. Nevertheless, future applications for designing new stems reflecting the characteristics of a specific population are promising. Additionally, by simplifying the process through coding, efficient analyses can be conducted using this streamlined platform.

## 5. Conclusions

Among femoral components, comprehensive results exhibited Fit to be the most suitable for the study population who possess a narrower distal canal in terms of implant design in the coronal plane. Accolade II also offered comparable results while also providing the widest coverage through adequate high offset options. That is, an optimal design for the population should possess a slimmer distal ML width and should offer high offset options with a reduced CCD angle to provide wider coverage for anatomical variations.

## Figures and Tables

**Table 1:** Demographics of the sample population included in the study.

Patients, *n*	742
Men (%)	313 (42.2%)
Women (%)	429 (57.8%)
Average age ± SD (range), years	63.98 ± 14.29
Average BMI ± SD (range), kg/m^2^	23.73 ± 3.17
Preoperative diagnosis, *n*	
ONFH	147 (42.9%)
Secondary osteoarthritis	68 (19.8%)
Primary osteoarthritis	59 (17.2%)
Femur neck fracture	55 (16.0%)
Rheumatoid arthritis	7 (2.0%)
Intertrochanteric nonunion	5 (1.5%)
DDH sequelae	2 (0.6%)

**Table 2:** Five single-tapered wedge proximal fit femoral stems included in the study.

Stem	Manufacturer	Available Conventional Stem Sizes
Tri-lock^®^	DePuy Orthopaedics	10 (#1–#9)
Taperloc^®^	Biomet Orthopedics	17 (#4–#20)
Anthology^®^	Smith and Nephew Inc.	12 (#1–#12)
Accolade II^®^	Stryker Orthopaedics	11 (#1–#11)
Fit^®^	Corentec	12 (#1–#12)

## Data Availability

The datasets used and analyses are available from the corresponding author upon reasonable request.
